# Austrian consensus statement on the diagnosis and management of hypertrophic cardiomyopathy

**DOI:** 10.1007/s00508-024-02442-1

**Published:** 2024-10-01

**Authors:** Nicolas Verheyen, Johannes Auer, Nikolaos Bonaros, Tamara Buchacher, Daniel Dalos, Michael Grimm, Agnes Mayr, Anna Rab, Sebastian Reinstadler, Daniel Scherr, Gabor G Toth, Thomas Weber, David K. Zach, Marc-Michael Zaruba, Daniel Zimpfer, Peter P Rainer, Gerhard Pölzl

**Affiliations:** 1 Division of Cardiology, Department of Internal Medicine, Medical University of Graz, Auenbruggerplatz 15, 8036 Graz, Austria; 2 Department of Internal Medicine 1 with Cardiology and Intensive Care, St. Josef Hospital Braunau, Braunau, Austria; 3 Paracelsus Medical University Salzburg, Salzburg, Austria; 4 Department of Cardiac Surgery, Medical University of Innsbruck, Innsbruck, Austria; 5 Department of Internal Medicine and Cardiology, Klinikum Klagenfurt, Klagenfurt, Austria; 6 Department of Cardiology, University Clinic of Internal Medicine II, Medical University of Vienna, Vienna, Austria; 7 University Clinic of Radiology, Medical University of Innsbruck, Innsbruck, Austria; 8 Department Internal Medicine I, Kardinal Schwarzenberg Klinikum, Schwarzach, Austria; 9 Department of Cardiology and Angiology, Medical University Innsbruck, Anichstraße 35, 6020 Innsbruck, Austria; 10 Department Innere Medizin II, Cardiology and Intensive Care Medicine, Klinikum Wels-Grieskirchen, Wels, Austria; 11 Department of Cardiac Surgery, Medical University of Vienna, Vienna, Austria; 12 BioTech Med, Graz, Austria; 13 Department of Medicine, St. Johann in Tirol General Hospital, St. Johann in Tirol, Austria

**Keywords:** Hypertrophic phenotype, Left ventricular outflow tract obstruction, Sudden cardiac death, Phenocopies, Heart failure

## Abstract

Hypertrophic cardiomyopathy (HCM) is the most common inherited heart disease that is characterized by left ventricular hypertrophy unexplained by secondary causes. Based on international epidemiological data, around 20,000–40,000 patients are expected to be affected in Austria. Due to the wide variety of clinical and morphological manifestations the diagnosis can be difficult and the disease therefore often goes unrecognized. HCM is associated with a substantial reduction in quality of life and can lead to sudden cardiac death, especially in younger patients. Early and correct diagnosis, including genetic testing, is essential for comprehensive counselling of patients and their families and for effective treatment. The latter is especially true as an effective treatment of outflow tract obstruction has recently become available in the form of a first in class cardiac myosin ATPase inhibitor, as a noninvasive alternative to established septal reduction therapies. The aim of this Austrian consensus statement is to summarize the recommendations of international guidelines with respect to the genetic background, pathophysiology, diagnostics and management in the context of the Austrian healthcare system and resources, and to present them in easy to understand algorithms.

## Introduction

The European Society of Cardiology (ESC) published the first international guidelines on cardiomyopathies, which also include an update on the 2014 ESC guidelines on hypertrophic cardiomyopathy (HCM), in 2023 [1, 2]. In 2020 the American College of Cardiology (ACC) and American Heart Association (AHA) Joint Committee issued guidelines on HCM [3]. Important specific aspects of HCM, such as imaging or physical activity, are specifically addressed in further international recommendations and position statements [4–8]. It is the aim of this Austrian consensus statement to aggregate the most important recommendations of international guidelines in the context of the Austrian healthcare system and resources. Austria is a high-income country with a healthcare system provided with high-level operational and human resources. Due to compulsory health insurance, more than 99% of the Austrian population have full and unrestricted access to all healthcare system services. All diagnostic methods and treatments for HCM including septal reduction therapy (SRT) are available at different tertiary care and HCM expert centers throughout Austria. Therefore, the recommendations issued in the following are applicable to all Austrian residents who are holders of a valid health insurance.

## Definition

Characteristic features of HCM include an end-diastolic left ventricular (LV) wall thickness ≥ 15 mm at any segment not explained by loading conditions [1, 3]. In patients with confirmed disease-causing mutation or in relatives of patients with confirmed HCM, a wall thickness ≥ 13 mm is indicative of HCM [8, 9].

According to the 2020 AHA guidelines, HCM is defined as a “disease state in which morphologic expression is confined solely to the heart and is characterized predominantly by LV hypertrophy (LVH; wall thickness ≥ 15 mm) in the absence of another cardiac, systemic, or metabolic disease capable of producing the magnitude of hypertrophy evident in a given patient and for which a disease-causing sarcomere (or sarcomere-related) variant is identified, or genetic etiology remains unresolved” [3]. By contrast, in the ESC guidelines on HCM and the 2023 ESC guidelines on cardiomyopathies, HCM is defined by a wall thickness ≥ 15 mm in one or more LV myocardial segments that is not explained solely by loading conditions, thereby including both sarcomeric and non-sarcomeric mutations, such as other genetic disorders, genetic syndromes and non-genetic disorders [1, 2]. Thus, the ESC definition alludes to a “HCM phenotype” covering both sarcomeric HCM and so-called HCM phenocopies. HCM phenocopies include inherited metabolic and neuromuscular diseases, chromosomal abnormalities and cardiac storage diseases, such as cardiac amyloidosis, among others. This consensus document focuses on sarcomeric HCM following the AHA definition, although HCM phenocopies are covered as differential diagnoses in a dedicated chapter and diagnostic algorithm (see Sect. 6.7).

A systematic diagnostic approach is warranted in patients with a HCM phenotype to confirm sarcomeric HCM and to exclude HCM phenocopies.

## Epidemiology

A variety of studies from different countries across different ethnicities has reported a prevalence of 0.16–0.23% for unexplained LV thickening in adult populations [10–14]. Of note, a large proportion of carriers of HCM-causing mutations are phenotype negative, i.e., they do not develop LV hypertrophy and remain clinically silent [15]. Combining prevalence estimates for both HCM phenotype positive and HCM genotype positive individuals, a prevalence of up to 0.6% has been hypothesized [16]. Based on these considerations it is estimated that more than 1 million individuals in Europe, 750,000 in America, and 20,000–40,000 in Austria may be affected by HCM [17]. HCM is largely underdiagnosed [18]. From this it can be estimated that only approximately 2500 individuals in Austria are clinically diagnosed. Currently, HCM is identified with increasing frequency in all age groups [19]. Although HCM is mostly inherited according to an autosomal dominant pattern, the majority of studies have reported higher rates in men, most likely because women are diagnosed less commonly than men [20]. Other factors that may explain the higher rate in men might include biases in screening and genetic and/or hormonal modifiers of phenotypic expression [2, 3].

HCM is characterized by variable expressivity (i.e., extent of disease manifestation) and penetrance. The clinical spectrum, even within families carrying similar mutations, can range from asymptomatic cases to clinically apparent disease during early childhood. Individuals with HCM may suffer from heart failure (HF) due to LV outflow tract obstruction (LVOTO) and/or structural heart disease, atrial fibrillation (AF) with increased risk of thromboembolism and ventricular arrhythmias potentially leading to sudden cardiac death (SCD) [17]. Approximately 30–40% of HCM patients will experience adverse events among referral-based cohorts [3]. Predictors of adverse events in patients with HCM include confirmation of a pathogenic sarcomeric mutation [21, 22], diagnosis at a young age [23], myocardial bridging [24], extensive late gadolinium enhancement (LGE) by cardiac magnetic resonance imaging (CMRI) [25, 26], impaired left (LV) and right ventricular (RV) function [27], excessive myocardial thickening [28, 29], history of syncope [30], LV apical aneurysm formation [31], non-sustained ventricular tachycardia (NSVT) according to outpatient monitoring [32] and LVOTO with a peak gradient of at least 30 mm Hg [33].

LVOTO is present in up to 75% of patients with HCM referred to tertiary care centers [34], while the prevalence of LVOTO in the general HCM population is unknown. LVOTO is an important determinant of symptoms and is associated with adverse outcomes [35]. While HCM patients without LVOTO have a 1.6% annual risk of developing New York Heart Association (NYHA) grade III/IV HF, this risk increases to 3.2% in the presence of provocable obstruction ≥ 30 mm Hg, and to 7.4% in case of significant resting LVOTO ≥ 30 mm Hg [17].

Prediction of outcomes in individual patients is limited by the large number and diversity of HCM-associated variants. Contemporary treatment options and interventions that target adverse disease pathways have significantly reduced HCM-associated morbidity and mortality. The estimated annual mortality rates have declined from 6% to 0.5% [36–38]. A major contributor to mortality reduction are SCD risk stratification strategies that are mainly based on a number of noninvasive risk markers and the use of implantable cardioverter-defibrillators (ICD) in individuals at the greatest risk of SCD. The SCD reduction has shifted morbidity and mortality to HF, which is currently the focus of established and future therapeutic strategies. In addition, the advent of effective management options for major HCM-related complications including LVOTO has significantly improved the clinical course and may result in substantially reduced morbidity and mortality. Overall, the diagnostic and therapeutic achievements established over the past decades have enhanced the likelihood of achieving a normal life expectancy with a good quality of life [17].

## Pathophysiology

At the macroscopic level, HCM is characterized by hypertrophied, small, stiff ventricles with impaired systolic and diastolic performance despite preserved, often even hyperdynamic (i.e., > 70%) LV ejection fraction (LVEF). Light microscopy reveals individual myocyte hypertrophy. Myocardial fiber disarray forming chaotic intersecting bundles is the pathognomonic abnormality, as opposed to linear parallel arrays seen in healthy individuals. Although fiber disarray is also noted in other diseases, the percentage of the myocardium occupied by disarray is higher in patients with HCM [39, 40]. Interstitial and perivascular fibrosis, which may affect as much as 14% of the myocardium in patients who die suddenly, is another frequent finding in HCM [41]. Fiber disarray and fibrosis are thought to predispose to electrical re-entry and sudden death [42].

Fibrosis and hypertrophy decrease the LV chamber compliance and cause diastolic dysfunction and exercise intolerance [43]. Although epicardial coronary arteries are dilated, narrowing of the intramural penetrating coronary arteries due to arteriolar intimal and medial hyperplasia is frequent. These constrictions are thought to contribute to ischemia in HCM [44]. Also, the prevalence of myocardial bridging is higher in adults with HCM compared to the average adult population [45].

The phenotypic expression includes a myriad of asymmetric and symmetric patterns of hypertrophy that are highly variable even among first-degree relatives.

1. Asymmetric hypertrophy at the confluence of the basal anterior septum with the contiguous anterior free wall (localized subaortic bulge) resulting in sigmoid morphology [46, 47] is the most common form (70%). Asymmetric septal hypertrophy is frequently associated with LV outflow obstruction and with anterior movement of the mitral valve.
2. The second most frequent form of asymmetric septal HCM is hypertrophy of the medial inferior interventricular septum. Septal thickening that occurs predominantly towards the middle segment of the LV results in a C-shaped morphology (reverse curve phenotype) and is associated with the presence of a disease-causing mutation in a sarcomeric gene in 80% [48]. Marked mid-ventricular septal hypertrophy along with a significant decrease in ventricular volume due to narrowing of the cavity can cause ventricular arrhythmia, myocardial necrosis and systemic embolism secondary to dynamic mid-ventricular obstruction associated with apical aneurysm [49]. Contrast-enhanced CMRI has demonstrated that the aneurysm rim in these patients is composed predominantly of fibrosis that extends from the aneurysm rim into the septum of the free wall and serves as a nidus for ventricular tachycardia [47].
3. Apical HCM (5–25%) shows predominantly apical distribution of hypertrophy with a characteristic spade-like configuration of the LV cavity and is usually associated with giant inverted anterolateral T‑waves on the electrocardiogram (ECG) [47]. Although apical hypertrophy is reported to have a better long-term prognosis than other forms of HCM, arrhythmic events are possible, and one third of patients can develop ventricular tachyarrhythmia.
4. A HCM with symmetric hypertrophy requires a detailed differential diagnostic delineation from other entities that present with diffuse hypertrophy of the myocardium, such as hypertensive cardiomyopathy, amyloidosis, Fabryʼs disease and athlete’s heart (see also Sect. 6.7; [50]). A minor percentage of patients show an increase in ventricular width limited to small and focal areas of the ventricle, usually confined to one or two segments. This can also be the clinical presentation of early stages of the disease [50]. Of the patients with HCM one third show a hypertrophied right ventricle which can involve all three segments, although a conspicuous proportion demonstrates RV hypertrophy that is confined to the segments adjacent to the septum [51].

### Left ventricular outflow tract obstruction

In the vast majority of cases LVOTO is caused by systolic anterior motion (SAM) of the mitral valve and mitral-septal narrowing or contact [41]. At least three principal mechanisms are responsible for LVOTO/SAM:

1. Septal hypertrophy with narrowing of the LV outflow tract (LVOT) leading to abnormal blood flow vectors that dynamically displace the mitral valve leaflets anteriorly.
2. Anatomic alterations of the mitral valve and apparatus, including longer leaflets as well as anterior displacement of the papillary muscles and mitral valve apparatus which make the valve more susceptible to abnormal blood flow vectors [3].
3. A hyperdynamic LVEF as a prerequisite to the induction of drag forces on the mitral valve. Importantly, LVOTO is dynamic and sensitive to ventricular load and contractility. Even a subtle increase in myocardial contractility or decrease in preload or afterload can lead to large variations in LVOT gradients and obstruction [52]. Spontaneous variability in the LVOT gradient can occur with irregular heartbeat (e.g., AF, extrasystoles), daily activities, food and alcohol intake or even with quiet respiration [53].

Finally, LVOTO develops as a result of sequential hemodynamic steps involving the mitral valve and its spatial relationship with the contracting ventricular septum during systole. At early systole, the mitral valve is swept towards the septum by the pushing force of flow, referred to as the drag force [54]. Once there is a significant attack angle of local vector flow on the posterior surface of the mitral valve, ventricular afterload increases [55]. Venturi forces become relevant and, by way of an amplifying feedback loop promote SAM further, thus narrowing the space between the mitral leaflet tip and the ventricular septum towards the end of systole [56].

Changes in the mitral valve and subvalvular apparatus are frequently encountered in patients with LVOTO. Septal bulge, large mitral leaflets that are anteriorly positioned in the LV cavity because of anterior displacement of the papillary muscle and residual proportions of the leaflets that extend past the coaptation point and protrude into the LVOT are anatomic features that expose the mitral valve to the hydrodynamic effects of flow, thus predisposing it to SAM [57, 58]. Chordal slack plays a permissive role and is necessary for SAM to occur as leaflets would be tethered without it [41]. Conversely, shortened chordae can pull the valve into the LVOT. Congenital anomalous insertion of the papillary muscle directly into the mitral valve without interposition of chordae is occasionally responsible for mid-ventricular muscular obstruction [36, 59]. Therefore, a thorough understanding of the anatomy and the underlying pathomechanism of the disease is critical for planning invasive treatment [36, 59].

Mid-cavity obstruction affects approximately 10% of patients with HCM and independently predicts the development of apical aneurysms, arrhythmic events and HCM-related death [60]. An RV obstruction in adults with HCM has been insufficiently investigated to provide a gradient cut-off and prevalence data.

## Diagnosis

Clinical evaluation of HCM is indicated in patients symptomatic with HF, cardiac arrest, arrhythmias and incidental findings such as detection of a heart murmur by routine clinical examination or abnormalities in the routine 12-lead ECG. Screening for HCM is indicated in relatives of patients with confirmed HCM.

**Fig. 1:**
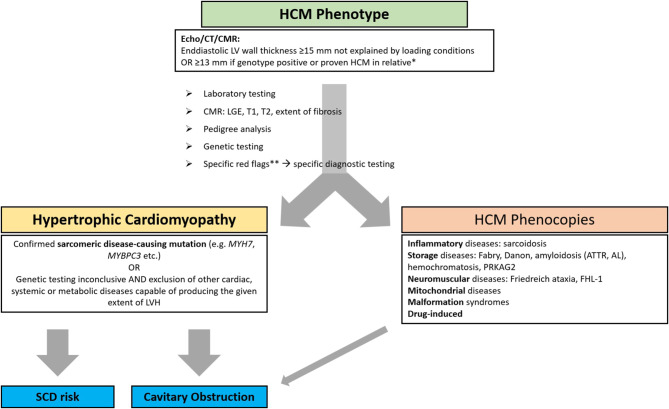
Diagnostic algorithm for hypertrophic cardiomyopathy. * In patients with resistant hypertension (defined as inadequate blood pressure control despite 3 antihypertensives including a diuretic): ≥ 17 mm measured by cardiac magnetic resonance imaging [9]. ** As comprehensively described in Sect. 6.7 and Table 1. *AL* light chain amyloidosis, *ATTR* transthyretin amyloidosis, *CMR* cardiac magnetic resonance imaging, *CT* computed tomography, *echo* echocardiography, *LGE* late-gadolinium enhancement, *LV* left ventricular, *LVH* left ventricular hypertrophy, *SCD* sudden cardiac death. (Adapted from [1–3, 7, 8, 61])

In a patient with an HCM phenotype, a systematic diagnostic work-up is required to confirm sarcomeric HCM and to rule out HCM phenocopies as depicted in Fig. 1. Besides standard cardiological assessments (clinical examination, 12-lead ECG and basic laboratory testing), the work-up comprises extended laboratory testing (including natriuretic peptides, iron status, free light chains in serum and urine, and testing for Fabry’s disease), focused transthoracic echocardiography (TTE), CMRI with contrast application, a pedigree analysis, genetic testing and assessment of red flags for HCM phenocopies. The diagnosis of HCM is confirmed if a disease-causing mutation in a sarcomeric gene is identified. If genetic testing is inconclusive, HCM phenocopies must be ruled out to confirm the diagnosis of HCM (see also Sect. 6.7 and Table 1).

**Table 1:** Differential diagnoses in patients with HCM phenotype—Cardiac and non-cardiac red flags (adapted from [1, 2, 5–7])

Disease	Non-cardiac red flags	Typical ECG findings	Typical echocardiography findings	Typical CMRI findings
HCM	Age < 40 years Positive family history of HCM or sudden cardiac death	Inferolateral T wave inversion ST-segment depression Abnormal Q waves Giant T wave inversion in apical involvement	Asymmetric LVH ≥ 15 mm septal/posterior wall thickness ratio > 1.3 apical hypertrophy regional and global abnormalities in GLS co-locating with regions of LVH Varying degrees of diastolic dysfunction SAM of mitral valve and LVOT obstruction	Patchy mid-wall LGE in hypertrophied areas LGE in ventricular insertion points
Hypertensive heart disease	Resistant arterial hypertension, i.e., inadequate blood pressure control despite 3 antihypertensives including a diuretic	Normal 12-lead ECG or isolated increased voltage without repolarization abnormality Prolonged QTc duration	Concentric LV hypertrophy initially reduction of GLS at basal septum Regression of LVH over 6–12 months with tight systolic blood pressure control (< 130 mm Hg)	Patchy LGE in mid-myocardium and epicardium Increased ECV fraction in some patients
Athlete’s heart	High intensity/competitive sport activity in patient history	Sinus bradycardia LVH Early repolarization First degree or Wenckebach AV block Ectopic atrial or junctional rhythm	Balanced 4 chambers dilation Normal diastolic function Normal left ventricular strain	LGE absent except occasionally at the RV insertion points Normal ECV
Amyloidosis	Bilateral carpal tunnel syndrome Ruptured biceps tendon Lumbar spinal stenosis Polyneuropathy Dysautonomia Skin bruising Macroglossia Deafness	Low QRS voltages relative to LV wall thickness Conduction abnormalities Pseudoinfarct pattern	Reduced GLS global longitudinal with apical sparing Bi-atrial dilation Thickening of valve leaflets and interatrial septum Granular sparkling of myocardium Restrictive LV filling pattern RV hypertrophy Pericardial effusion	Subendocardial and/or transmural LGE Prolonged native myocardial T1 relaxation time Increased ECV fraction
Anderson Fabry	Age < 40 years Positive family history of heart failure or hemodialysis No male-to-male transmission in pedigree Neuropathic pain Angiokeratomas Albuminuria Progressive renal failure Cornea verticillata Hypohidrosis, heat/cold and exercise intolerance Gastrointestinal symptoms (nausea, vomiting, non-specific abdominal pain, constipation, diarrhea) Hearing loss, tinnitus, vertigo	Short PR interval, pre-excitation Atrioventricular blocks in adult patients Bradycardia Chronotropic incompetence LVH	LVH with normal systolic function Hypertrophy of papillary muscles mitral and aortic valve thickening with mild to moderate regurgitation Reduced GLS	Basal-inferolateral LGE Low native T1 (caution with pseudonormalization in areas affected by fibrosis) High focal/global T2

*CMRI* cardiac magnetic resonance imaging, *ECV* extracellular volume, *ECG* electrocardiogram, *GLS* global longitudinal strain, *HCM* hypertrophic cardiomyopathy, *LGE* late gadolinium enhancement, *LVH* left ventricular hypertrophy, *LVOT* left ventricular outflow tract

### Clinical examination and patient history

Clinical assessment in patients with suspected HCM or relatives of HCM patients in the context of family screening is mandatory. It includes the assessment of central and peripheral congestion and auscultation with particular alertness regarding cardiac murmurs. Obstructions of the LV cavity are auscultable as mid to late systolic murmurs over Erb’s point radiating towards the right sternal edge and the apex, with increasing volume during provocative maneuvers or exercise testing (see also Sect. 5).

The patient interview includes assessment of exertional dyspnea with classification using the NYHA functional class, angina pectoris, syncope or presyncopes including precipitating factors, and palpitations. Exertional dyspnea due to cavity obstruction typically worsens after heavy meals, alcohol consumption and exsiccosis. The family history assessment covers at least three generations and includes diagnoses of HF, heart transplantation, implantation of a pacemaker or defibrillator, SCD, and red flags for HCM phenocopies (see also Sect. 6.7 and Table 1), such as chronic kidney disease requiring hemodialysis, stroke at a young age, skeletal muscle weakness, diabetes mellitus and deafness. A pedigree should be drawn to enable conclusions to be drawn on genetic traits.

### Electrocardiography

#### 12-lead ECG

A 12-lead ECG is mandatory in every patient with suspected HCM during initial evaluation and routine follow-up, and in relatives of HCM patients during family screening. Given its limited diagnostic accuracy, the 12-lead ECG must be interpreted in the context of the patient’s symptoms, history, and cardiac imaging.

Unremarkable ECG findings are present in less than 10% of HCM patients and are associated with a milder clinical phenotype, although HCM cannot be ruled out based on a normal ECG [62]. Non-voltage ECG changes favoring the presence of HCM in patients with an HCM phenotype include pathological Q waves, ST-segment depression or elevation, and T‑wave inversions. The sensitivity varies largely due to heterogeneity of reported HCM cohorts but rarely exceeds 50% [63]. According to the ESC guidelines the following parameters are considered relatively specific of HCM.

1. Giant T‑wave inversion (> 10 mm) in precordial or inferolateral leads indicates LV apex involvement.
2. Abnormal Q‑waves (≥ 40 ms duration and/or ≥ 25% of R‑wave and/or ≥ 3 mm depth at least in 2 parallel leads, except aVR) with positive T‑waves indicate an asymmetric LV hypertrophy pattern. Q‑waves with abnormal duration of ≥ 40 ms also indicate myocardial fibrosis.
3. Sustained ST-segment elevation in the lateral leads associated with apex aneurysm and myocardial scarring in patients with apical or distal septal hypertrophy.

The ECG voltage criteria, such as the Sokolow-Lyon index or Cornell criteria, are almost 100% specific for LVH; however, the sensitivity of ECG voltage criteria for LVH is poor, as only 10% of the general population and 40% of patients in HCM cohorts fulfil ECG criteria for LVH [63, 64]. An ECG can help to differentiate HCM from HCM phenocopies such as cardiac amyloidosis where the QRS amplitude is low relative to the extent of wall thickening observed in cardiac imaging (see also Sect. 6.7 and Table 1). Massive LV hypertrophy (Sokolow-Lyon index ≥ 50 mV) can indicate storage diseases such as Pompe disease or Danon disease [2].

#### Outpatient Holter ECG

A 24/48‑h outpatient Holter ECG is mandatory in HCM patients to assess the risk of SCD. Moreover, it is recommended in patients who report symptoms such as palpitations or lightheadedness to detect corresponding arrhythmias, particularly ventricular tachyarrhythmias or AF. In patients with clinically suspected arrhythmias and inconclusive Holter ECG readings, prolonged outpatient ECG monitoring from 72 h to 7 days may be considered, as well as wearable event monitors (see also Sect. 7.1) or implantable monitors [3].

### Imaging

#### Echocardiography

The use of TTE is mandatory in any patient with suspected HCM. Beyond standard echocardiographic measures of cardiac structure and function TTE in a patient with an HCM phenotype incorporates specific assessments of hypertrophy, LV filling pressures, the mitral valve and its apparatus, and cavity obstruction as summarized in Table 2.

**Table 2:** Echocardiography-derived parameters for the assessment of patients with hypertrophic cardiomyopathy (adapted from [2–4])

	Echocardiographic parameter	Threshold for abnormal value
Hypertrophy	LV wall thickness: basal, mid and apical level^a^	≥ 15 mm
	Septal hypertrophy shape	Sigmoid, reverse curve, apical, symmetric
	RV wall thickness: free wall, anterior wall	≥ 7 mm
Diastolic function—filling pressures	LAVI (method of disks)	> 34 ml/m^2^
	Septal e‘	< 7 cm/s
	Lateral e‘	< 10 cm/s
	E/A	≥ 2
	E/e’ ratio	> 14
	Peak TR velocity	> 2.8 m/s
	Ar duration—A duration	≥ 30 ms
Cavity obstruction: gradients assessed at rest and during provocation	LVOT obstruction	SAM of mitral valve Late systolic obstruction signal Peak LVOT gradient ≥ 30 mm Hg Abnormal insertion of papillary muscle
	Mid-cavity obstruction	Late systolic obstruction signal Peak mid-ventricular gradient ≥ 30 mm Hg Apical aneurysm (contrast agent)
	Right ventricular obstruction	Late systolic obstruction signal Cut-off unclear

*A* peak late transmitral filling velocity, *A duration* duration of late transmitral filling, *Ar duration* duration of pulmonary venous atrial reversal flow, *E* peak early transmitral filling velocity, *e‘* peak early mitral annular velocity, *IVRT* isovolumetric relaxation time, *LAVI* left atrial volume index, *LV* left ventricular, *LVOT* left ventricular outflow tract, *RV* right ventricle, *SAM* systolic anterior motion, *TR* tricuspid regurgitation

^a^≥ 13 mm in case of familiar HCM or a confirmed disease-causing mutation

Maximum wall thickness of any LV segment, preferably acquired in parasternal axes, is the essential measure of hypertrophy in HCM. The morphology of septal hypertrophy (sigmoid, reverse curve, apical, symmetric) is best assessed in apical views. Certain patterns of myocardial hypertrophy may allude to specific HCM phenocopies (see also Sect. 6.7 and Table 1).

**Fig. 2:**
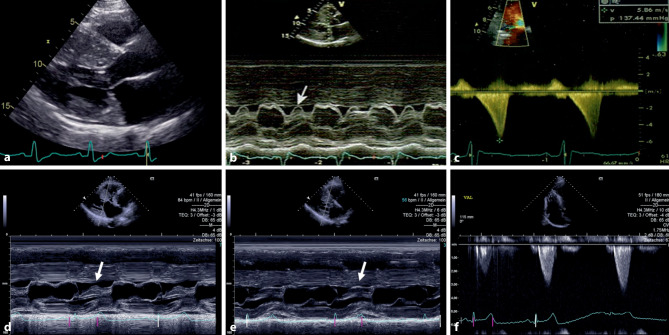
Echocardiographic features of left ventricular outflow tract obstruction. **a**–**c** patient with resting obstruction, **d, e** patient with provocable obstruction. **a** B-mode in parasternal long axis showing complete SAM at late systole. **b** M-mode in parasternal long axis showing complete SAM (*arrow*). **c** Dagger-shaped obstruction signal derived from continuous wave Doppler in apical 5‑chamber view. Bottom: patient with provocable obstruction. **d** M-mode in parasternal long axis showing incomplete SAM at rest (*arrow*). **e** M-mode in parasternal long axis showing incomplete SAM during Valsalva maneuver (*arrow*). **f** Obstruction signal derived from continuous wave Doppler in apical 5‑chamber view with increasing gradient during Valsalva maneuver. *SAM* systolic anterior motion

**Fig. 3:**
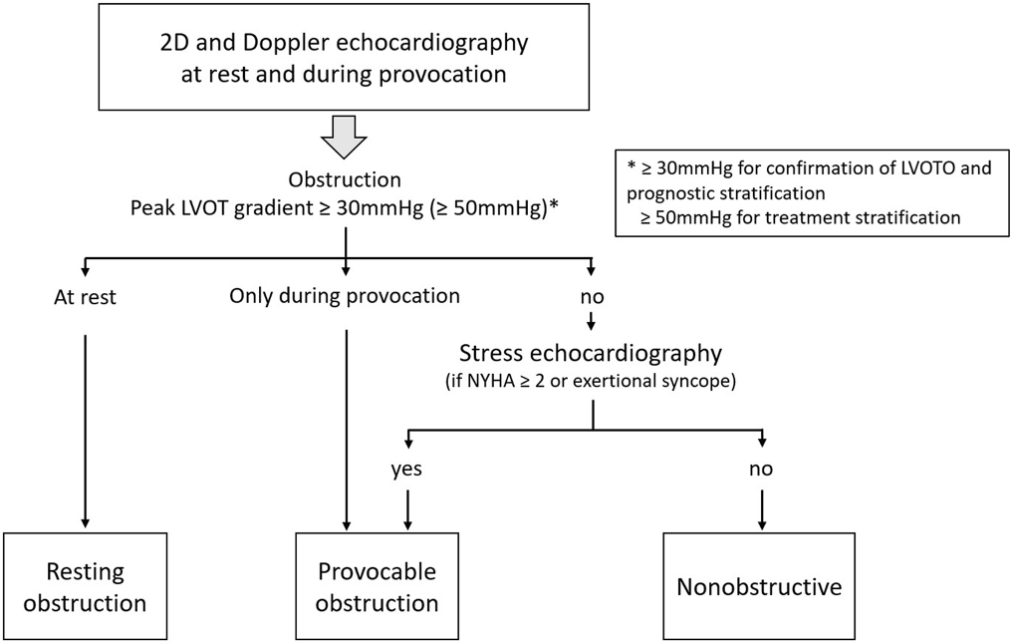
Diagnosis of left ventricular outflow tract obstruction by echocardiography in patients with hypertrophic cardiomyopathy or hypertrophic cardiomyopathy phenotype. *LVOTO* left ventricular outflow tract obstruction, *NYHA* New York Heart Association functional class (adapted from [1])

With respect to LVOTO, the presence and extent of SAM of the mitral leaflets should be assessed either in B‑mode or applying M‑mode in the parasternal long or short axis. The SAM is defined as motion of an entire leaflet or at least a leaflet tip towards the ventricular septum during systole. It is described as incomplete if there is no septal contact and as complete in the case of septal contact (see Fig. 2). The presence of SAM should prompt thorough assessment of LVOTO including provocative maneuvers as shown in Fig. 3. Assessment of the late systolic distance between the mitral leaflet tip and the ventricular septum (TIS) at rest in the apical 3‑chamber view can also help to assess the likelihood of LVOTO (TIS ≤ 9 mm: LVOTO likely; TIS > 14 mm: LVOTO unlikely) [65].

The LVOT gradients should be measured at rest and during provocation by placing the continuous wave (CW) Doppler beam through the narrowest space between the mitral valve and ventricular septum in the apical 5‑chamber and 3‑chamber views. The LVOTO signal increases towards the end of systole, leading to a typical late systolic, dagger-shaped obstruction signal. Provocative maneuvers are crucial for unmasking relevant intracavity obstruction. Gradients should be assessed at rest and during Valsalva maneuver in semi-supine, sitting and standing positions. More sophisticated provocative maneuvers include the squat-to-stand maneuver (standing up after 10 s in a squat position) and post-exercise gradient following exercise testing or stress echocardiography using a treadmill or bicycle. These are indicated in symptomatic patients. Dobutamine stress echocardiography is not recommended because it is not physiological and can also lead to false positive results due to direct inotropic effects of dobutamine that induce intracavity obstruction [1, 66]. Additionally, nitrite inhalation may be used in experienced hands. As the postprandial state is associated with an increase in LVOT gradients, reassessment of patients with suspected LVOTO and inconclusive echocardiography within 1 h after a conventional meal can deliver higher LVOT gradients und unmask significant LVOTO [67]. In some patients, the precise localization of obstruction and the differentiation between LVOTO and other causes of flow acceleration (e.g., aortic stenosis, mid-cavity obstruction) can be difficult. Here, application of pulsed-wave Doppler with alternating positioning of the sample volume can be helpful. If a subaortic membrane is suspected, TEE is indicated.

For the assessment of mid-cavity and RV obstructions, the mid-ventricular gradients should be measured at rest and during provocation using CW-Doppler in apical views. An apical aneurysm should be ruled out if a significant mid-ventricular gradient ≥ 30 mm Hg is detected. Visualization of an apical aneurysm can be improved by contrast echocardiography. Care must be taken to adapting the mechanical index when applying a transpulmonary contrast agent.

The assessment of RV obstruction can be difficult using TTE due to inadequate angulation or poor resolution. Depending on the anatomy, the parasternal short axis view, apical 4‑chamber view and subcostal view can be useful. The RV obstruction should be confirmed using invasive determination of gradients.

Primary abnormalities of the mitral valve and its apparatus, such as excessive leaflet length, anomalous papillary muscle insertion and displaced papillary muscles, are common in HCM and are considered part of its phenotypic expression. These abnormalities can incite mitral regurgitation either independently or as a secondary consequence of SAM of the mitral valve in HCM patients. Analogous to LVOTO, the severity of mitral regurgitation depends on factors influencing ventricular load and myocardial contractility. Consequently, significant mitral regurgitation may not become apparent without provocation for LVOTO following SAM of the mitral valve.

When planning myectomy TTE should be supplemented by TEE and CMRI to obtain information about the potential involvement of the subvalvular apparatus and the pattern and localization of hypertrophy, especially in cases of RV obstruction, where TTE has its limitations.

The TTE parameters required for the assessment of the indication of primary prophylactic ICD treatment include assessment of the left atrial diameter in parasternal long or short axis views, maximum LV end-diastolic wall thickness, maximum peak LVOT gradient, presence of an apical aneurysm and LVEF.

**Fig. 4:**
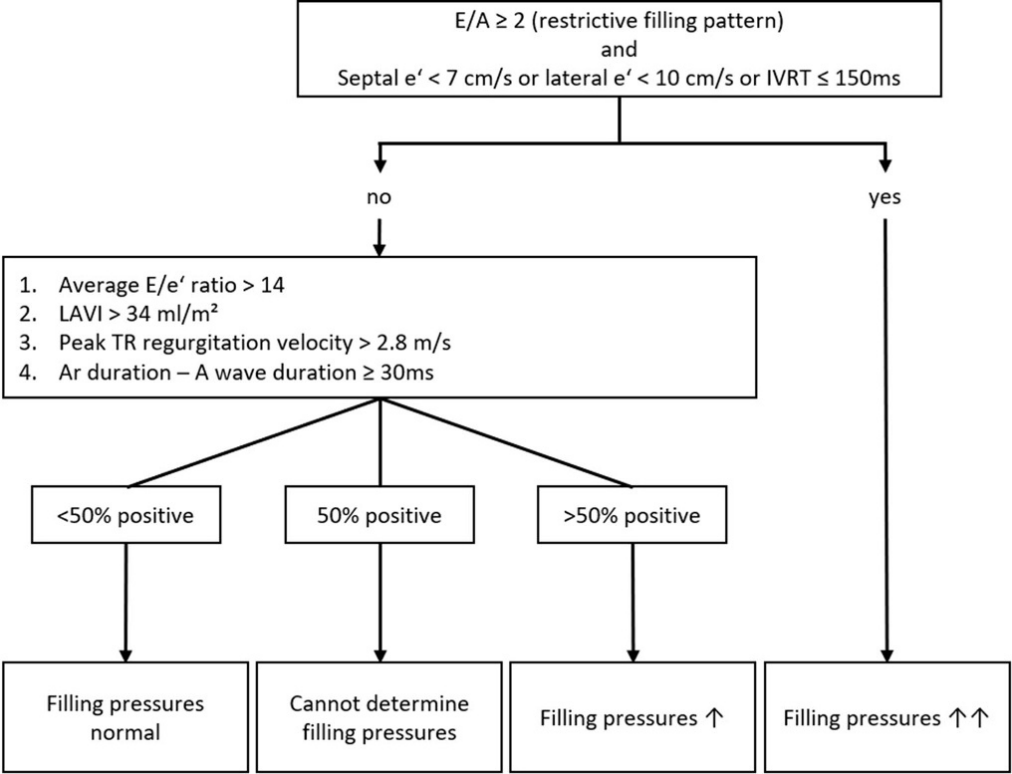
Assessment of left-sided filling pressures by echocardiography in patients with hypertrophic cardiomyopathy. *A* peak late transmitral filling velocity, *Ar* pulmonary venous atrial reversal flow, *E* peak early transmitral filling velocity, *e‘* peak early mitral annular velocity, *IVRT* isovolumetric relaxation time, *LAVI* left atrial volume index, *TR* tricuspid regurgitation (adapted from [2, 4])

Diastolic function is per definition impaired in a patient with HCM. Filling pressures are likely increased in cases of a restrictive LV filling pattern, i.e., a ≥ 2 ratio of early filling peak mitral velocity to late filling peak mitral velocity (A) and reduced e’ (either septal e’ < 7 cm/s or lateral e’ < 10 cm/s) or E‑wave deceleration time ≤ 150 ms [2, 4]. In HCM patients without a restrictive filling pattern, single echocardiographic indices of diastolic dysfunction show only a modest correlation with filling pressures. Rather, a HCM-specific composite of four echocardiographic indices is recommended, including the E/e’ ratio, left atrial volume index, pulmonary vein reversal velocity, and peak tricuspid regurgitation jet velocity. In the clinical routine, single criteria may not be available due to poor image quality, absence of tricuspid regurgitation or unreliability of e’, for instance in the presence of mitral annulus calcification. Filling pressures are considered elevated if more than 50% of the available parameters are abnormal and normal filling pressures are reported if more than 50% of the available parameters are normal. The LV filling pressure cannot be determined if 50% are normal and 50% are abnormal (Fig. 4).

#### Cardiac magnetic resonance imaging

The multiparametric approach to CMRI provides a comprehensive characterization of cardiac morphology, function and myocardial tissue in the HCM phenotype, fusing spatial, contrast and temporal resolution. While echocardiography is the primary imaging modality for the evaluation of patients with suspected HCM, CMRI not only offers synergistic effects but has certain advantages for diagnosis and risk stratification. With respect to the unique possibilities of tissue characterization, the established LGE technique enables reliable visualization of focal replacement fibrosis, the newer mapping techniques offer absolute quantification of magnetic tissue properties (T1, T2, and T2* relaxation times and extracellular volume, ECV) and thus the detection of diffuse myocardial texture changes [68]. Therefore, CMRI is the key for differentiating HCM from HCM phenocopies that are clinical conditions with similar morphology but different underlying etiology, such as amyloid or Anderson-Fabry disease. Moreover, CMRI enables accurate detection of the location and extent of LVH also in LV areas that can be blinded to echocardiography and can depict associated abnormalities such as SAM of the mitral valve and apparatus, subaortic flow turbulence/obstruction, and mitral regurgitation [69]. A polymorphic appearance that extends far beyond hypertrophy alone is a characteristic feature of HCM. Mild preclinical phenotypes such as increased ECV, inverted septal curvature, or narrow myocardial crypts represent early CMRI manifestations that may be helpful in differentiating it from athlete’s heart [70].

**Fig. 5:**
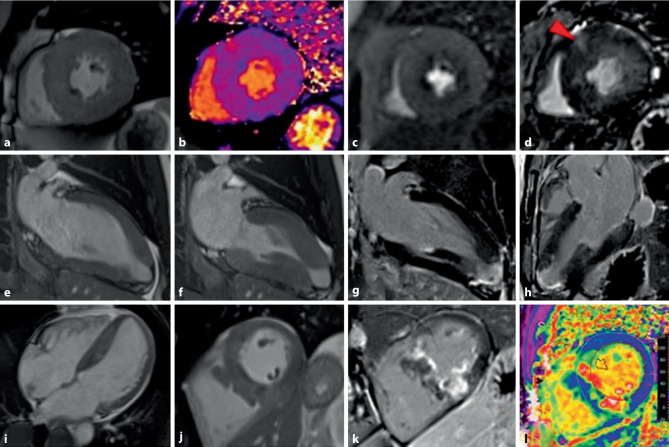
Features of hypertrophic cardiomyopathy in cardiac magnetic resonance imaging (CMRI). **a-d** patient 1, **e-h** patient 2, **i-l** patient 3. *Patient 1: a 52-year-old patient with a symmetrical form of hypertrophic cardiomyopathy (HCM).* **a** CINE (short axis view, end-diastolic): the interventricular septum measures a maximal thickness of 27 mm. **b** T1 mapping (short axis view) without contrast medium shows a moderate increase in T1 relaxation times indicative of diffuse interstitial fibrosis (T1 relaxation times septal of maximum 1063 ms on a 1.5T cardiac magnetic resonance imaging (CMRI) unit). **c** First-pass perfusion (short axis view) reveals resting subendocardial perfusion defects that may occur in HCM due to severe microvascular remodeling and dysfunction of small intramural arterioles. **d** On late gadolinium enhancement (LGE) sequence only a small focal anterior right ventricular insertion scar is evident (red arrowhead). *Patient 2: a 41-year-old patient with an asymmetrical apically distributed HCM form showing the typically spade-like left ventricular configuration with the formation of a small apex aneurysm.* **e, f** CINE (2 chamber view) in an end-diastolic phase (e) and an end-systolic phase (f) showing apically hypertrophied myocardial segments and formation of a small apical aneurysm. Apex aneurysms generally occur in only 2% of HCM patients, but are more common in patients with apical HCM (in 13%). HCM patients with apical aneurysms demonstrated a 4.7% annual rate of sudden cardiac death and 1.1% thromboembolic events/year [31]. **g, h** LGE sequences (**g**: 2 chamber view and **h**: three chamber view) show apical transmural fibrosis, the genesis of which is microangiopathy. Moreover, a small apex thrombus can be seen within the apex aneurysm. *Patient 3: a 49-year-old patient with an asymmetrically mid-ventricular septal distributed HCM form.* **i, j** CINE (**i**: 4 chamber view, end-diastolic) and CINE (**j**: short axis view, end-diastolic) measure an interventricular septum of maximal 22 mm width. **k** LGE (short axis view) shows pronounced replacement fibrosis in the hypertrophied septal segments. **l** Extracellular volume (ECV) map (short axis view) shows a local increase in ECV in the hypertrophied segments (ECV fraction in the septum up to 52%)

In addition, CMRI improves risk stratification as illustrated by novel CMRI-based risk markers (LV apical aneurysm, extensive LGE, and end-stage disease with systolic dysfunction) recently included in risk stratification algorithms for sudden death in HCM [3]. In vivo characterization of myocardial tissue by LGE and mapping techniques details the visualization and quantification of focal and diffuse myocardial fibrosis, respectively (see Fig. 5). About 60% of HCM patients show LGE, predominantly in the most hypertrophied segments. An extent of ≥ 15% LGE LV mass is associated with a significantly higher rate of SCD and is considered a key imaging feature in HCM risk stratification [71]. The use of T1 mapping as well as ECV determination enable the detection of diffuse fibrosis that may not be detected by LGE imaging and have been shown to independently predict adverse events in HCM [72].

The use of CMRI has also proven to be particularly useful for the definition of the LVOT anatomy in preoperative planning for invasive septum reduction. Longitudinal and perpendicular CINE imaging through the LVOT provide important anatomical details of the mitral valve allowing for optimal visualization of SAM and the subvalvular apparatus. In patients with obstruction at the mid-ventricular level and/or the RV outflow tract, MR images provide critical anatomical details for the planning of complex surgical procedures.

### Exercise testing

Exercise testing may be useful at the initial clinical evaluation to determine functional capacity and provide prognostic information. In symptomatic patients, exercise testing in combination with TTE can aid in unmasking LVOTO.

Cardiopulmonary exercise testing (CPET) is useful to objectify the physical capacity, with the most relevant parameters being peak oxygen consumption (peak VO 2), minute ventilation relative to CO_2_ production (VE/VCO_2_) slope, as well as aerobic and anaerobic threshold. Using CPET may be considered to quantify the severity of exercise limitation during evaluation for cardiac myosin inhibitor therapy or SRT, and to assess clinical response. The CPET is required in HCM patients with advanced HF during evaluation for heart transplantation [1].

### Heart catheterization and endomyocardial biopsy

Visualization of coronary arteries is mandatory in patients with HCM. A coronary CT scan can be performed to exclude coronary abnormalities such as coronary bridging, which can contribute to dyspnea, ventricular arrhythmias and sudden death. Coronary angiography is needed when alcohol septal ablation (ASA) or surgical myectomy are considered. In symptomatic patients and inconclusive non-invasive cardiac imaging, left and right heart catheterization may be considered to assess the severity of LVOTO at rest and with provocation (Valsalva and Brockenbrough maneuvers) and to measure LV filling pressures. An endomyocardial biopsy is required if HCM cannot be differentiated from HCM phenocopies by non-invasive testing. Electron microscopy can be useful if a storage or mitochondrial disease is suspected [1, 6].

### Genetic testing

HCM represents the most common genetic heart disease. In cases of a monogenic origin, HCM has an autosomal dominant pattern of inheritance. Given the possibility of de novo mutations, a genetic origin cannot be ruled out in the presence of a negative family history. HCM is characterized by variable expressivity, genotype-phenotype uncoupling, and variable penetrance of clinically overt disease [73]. Autosomal recessive and X-linked modes of inheritance have been described but are rare [74]. Currently, eight genes encoding contractile myofilament proteins of the cardiac sarcomere and Z‑band are considered as definitive HCM-associated genes [75].

Among patients with HCM, approximately 60% have a positive family history, and approximately 30–60% have a single identifiable pathogenic or likely pathogenic gene variant. Within this group, de novo mutations (i.e. sporadic HCM) are present in one third of patients. In a substantial proportion of patients with HCM, no monogenic cause can be identified, which includes a subgroup of up to 40% of patients without affected family members (non-familial HCM) [76].

Among patients with a pathogenic sarcomeric gene variant, the two most common genes are *beta myosin heavy chain 7* (*MYH7*) and *myosin-binding protein C3* (*MBPC3*), together representing > 90% of pathogenic variants [77], while other genes (*TNNI3, TNNT2, TPM1, MYL2, MYL3, ACTC1*) account for a small proportion of patients (1–5% each). Within these genes, a high number of variants have been recognized, the majority of which are private (i.e. unique to the individual family). Each offspring of an affected family member has a 50% chance of inheriting the variant [78]. The likelihood of developing clinical HCM is high in family members with a pathogenic variant; however, the age at which disease expression occurs and the expressiveness in a given individual vary.

The echocardiographic appearance may indicate an increased likelihood of sarcomeric-protein mutation in HCM: a reversed septal curvature causing a crescent-shaped cavity predicts gene-positive patients as compared with those with localized subaortic bulge and preserved septal curvature [48].

Specific genetic counselling and extended genetic testing (cascade genetic evaluation) is advised in patients with negative panel testing or non-pathogenic gene variants and high suspicion of a hereditary disease.

The precise mechanisms by which sarcomere variants result in a clinical phenotype are not yet fully understood. Mutant sarcomere genes trigger several myocardial changes, leading to hypertrophy and fibrosis, which ultimately result in small, stiff ventricles with impaired systolic and diastolic performance despite preserved LVEF [79]. From a metabolic viewpoint, mutations in sarcomeric proteins generally increase myofilament activation and result in myocyte hypercontractility and excessive energy use due to higher mitochondrial activity [80]. Mitochondrial impairment in the cardiac energy-sensing apparatus as well as alterations in calcium handling result in stimulation of signalling pathways that contribute to myocyte relaxation abnormalities and growth, with aberrant tissue architecture abnormalities such as myofibrillar disarray and myocardial fibrosis [81].

Recent data from clinical and network medical analyses as well as contemporary genetic studies, however, are challenging the monogenic hypothesis of HCM [82]. The “postmonogenic hypothesis” is based on various inconsistent findings: First, approximately half of patients with HCM do not demonstrate evidence of a monogenic cause. Second, a substantial proportion of HCM family members who carry the same pathogenic sarcomere mutation identified in a relative with a clinical diagnosis of HCM never develop phenotypic evidence of HCM. Third, certain morphologic features of the disease phenotype, e.g., changes in the mitral valve and subvalvular apparatus, vasculopathy involving the small intramural coronary arterioles, and the expanded interstitial (collagen) matrix and extracellular space, cannot be explained solely by mutated genes encoding proteins of the cardiac sarcomere [82]. These considerations place a new focus on possible novel interactions between acquired disease determinants and the genetic context to produce complex HCM phenotypes. In that respect, the ESC guidelines on cardiomyopathies emphasize the important role of arterial hypertension, diabetes and obesity as modulators of disease expression, particularly in HCM patients without monogenic disease [1].

### Differential diagnosis—HCM phenocopies

Several cardiomyopathies can present with an HCM phenotype and thereby mimic sarcomeric HCM, so-called HCM phenocopies. An HCM phenotype is defined in the ESC guidelines by non-adaptive severe LVH, i.e., an end-diastolic LV wall thickness ≥ 15 mm not explained by loading conditions [1, 2]. An LVH in sarcomeric HCM is usually asymmetric. Focal asymmetric hypertrophy of the basal anterior septum, defined as the ratio of septal:posterior wall thickness > 1.3 in a normotensive patient, is the most common pattern of LVH in HCM [5]. Variants (see Sect. 5) include a sigmoid septum, reverse septal curvature, concentric, and apical hypertrophy [5].

Even in the presence of normal LVEF, regional and global abnormalities in longitudinal strain colocating with regions of LVH (septum, apex) are often found [5]. Varying degrees of diastolic dysfunction and filling pressures are observed. Both SAM and LVOTO, either at rest or during provocation, are present in up to 75% of patients with HCM; however, SAM and LVOTO can also occur in both HCM phenocopies and adaptive LVH and thus are typical but not diagnostic for HCM. An LGE is noted in approximately half of patients with HCM and is commonly described as patchy and mid-myocardial within segments of maximum hypertrophy or the RV insertion points [5]. Moreover, abnormally prolonged T1 time and abnormally increased ECV fraction are often observed in HCM [83]. Other typical features of HCM are papillary muscle abnormalities (hypertrophy, displacement, direct leaflet insertion), myocardial crypts, mitral valve leaflet elongation, myocardial bridging and RV hypertrophy [3].

The HCM phenocopies can be caused by infiltrative diseases (e.g., amyloidosis), metabolic diseases (e.g., Fabry disease) and other rare diseases. Many HCM phenocopies are characterized by specific cardiac and non-cardiac red flags which should be assessed to guide the diagnostic pathway (see Table 1). The most common causes of adaptive LVH in adults are arterial hypertension, aortic stenosis, athlete’s heart and, rarely, subaortic membrane, all of which can infrequently cause severe LV hypertrophy ≥ 15 mm.

In arterial hypertension the LV remains one of the main target organs and echocardiographic measurements of structure and function provide prognostic information in this setting [84]. The LVH and hypertensive heart disease are the consequence of pressure overload, leading to concentric remodelling and hypertrophy. Depending on age, sex, duration, severity and treatment of hypertension, differing cellular and molecular events may underlie the evolution from a ventricle with concentric hypertrophy to a more dilated failing ventricle (often presenting as HF with reduced ejection fraction) or to a heavily fibrotic and non-dilated ventricle (presenting as HF with preserved ejection fraction) [84]. The majority of White patients with hypertensive LVH have a maximum interventricular septal thickness of < 15 mm [2]. Echocardiography-derived ventricular wall thickness ≥ 15 mm occurs in less than 5% of patients with mild-to-moderate arterial hypertension [85]. An LGE in the mid-myocardium and epicardium as well as diastolic abnormalities and left atrial dilation, can be present in both hypertension and HCM [2]. In a CMR-based study, the most accurate measure for separating HCM and hypertensive heart disease was LV maximum wall thickness ≥ 17 mm [9]. Finally, regression of LVH based on treatment and control of hypertension after 6–12 months is a strong argument supporting the diagnosis of hypertensive heart disease.

In athlete’s heart, obtaining a thorough history related to the duration, type and intensity of training is mandatory, simply because a lack of sufficient physical exercise excludes the diagnosis [86]. In most studies, athletes were compared to sedentary HCM patients. Therefore, a clear diagnosis in the “overlap” zone, where both factors are present, may be difficult to obtain [5]. Thus, assessment of family history of HCM and symptoms is mandatory in athletes with LVH. Typical ECG clues for athlete’s heart include sinus bradycardia, LVH, first-degree heart block, and AV block 2 type Wenckebach. An LVH attributable to (competitive) athlete’s heart is typically mild (11–13 mm in Caucasians, up to 15 mm in Black athletes and Caucasians with large body habitus) [87]. The TTE measurements of wall thickness taken from parasternal long-axis views must be made carefully to avoid inclusion of RV septal trabeculations and posterolateral chordal tissue [87]. At least some isometric stress during training should be present to explain LV wall thickening, while LV wall thickening in competitive endurance athletes should be accompanied by concomitant LV dilation, normal or accentuated LV diastolic function, symmetric LVH with only mild segmental variation, preserved or low normal (EF 45–55%) systolic function, and preserved myocardial LV systolic strain [87]. Among competitive athletes LVH develops as a function of myocyte enlargement with minimal fibrosis [88]. Finally, a competitive athlete will typically have excellent exercise capacity.

In cardiac amyloidosis similarities with HCM may include LVH (the mean septal and posterior wall thickness in the ATTR-ACT trial was 17 mm [89]), bi-atrial enlargement, evidence of diastolic function and elevated filling pressures [5] and RV hypertrophy [90]. Common echocardiographic findings include an abnormal global longitudinal strain (GLS) with an apical sparing (“cherry on top”) pattern [91]. In the majority the EF is preserved (≥ 50%) or mildly reduced (41–49%). Technetium-based bone scintigraphy in conjunction with a negative free light chain assessment has a high diagnostic accuracy for the diagnosis of cardiac ATTR amyloidosis [90].

Anderson-Fabry disease, caused by α‑galactosidase A deficiency, is a multisystemic X‑linked genetic disease (see Table 1). Thus, men are predominantly affected and experience the most severe clinical phenotype, while (heterozygous) women may constitute symptomatic or asymptomatic carriers [86]. Electrocardiographic keys include a short PR interval without a delta wave and a prolonged QRS interval, in addition to the presence of LVH [86]. Imaging findings include concentric, asymmetric or eccentric LVH, increased LV volume, gradual transformation to a more spherical LV shape, prominent papillary muscles, aortic dilation, and myocardial replacement fibrosis, which is often localized in the basal posterolateral wall, even with preserved LVEF. Longitudinal strain in the basal posterolateral wall may be reduced in early disease stages, and the basal inferolateral LV is thin in advanced disease. The diagnosis can be established by measuring α‑galactosidase A activity in leucocytes in male patients, and by genetic testing both in women and men.

Other diseases which can lead to an HCM phenotype include inflammatory diseases (e.g., sarcoidosis), storage diseases (e.g., Danon disease, hemochromatosis, PRKAG2), syndromal diseases (Friedreich ataxia, *FHL‑1*, malformation syndromes), mitochondrial diseases and drug-induced cardiomyopathy. Specific diagnostic work-up is indicated if indicative red flags are present and a previous diagnostic work-up has been inconclusive. Disease-specific red flags are extensively summarized in the ESC guidelines on cardiomyopathies [1].

Regarding LVOTO in cardiac diseases with LVH, it has been shown that LVOTO associated with SAM can be present in all cardiac diseases associated with LVH. In 2472 symptomatic adult patients with clinically diagnosed HCM and severe LVOTO who underwent surgical myectomy, 82% were eventually diagnosed with HCM, 11% with hypertensive heart disease, 1.3% with Fabry disease, 1% with amyloidosis, and 5% with concomitant myocarditis [92]. There were no significant differences in basal septal thickness and LVOT gradients across different etiologies. Especially in older patients with HCM, arterial hypertension (with or without LVOTO) affects up to 70%, making the presence of hypertension per se unreliable as a discriminator [93]. The coexistence of severe concentric LVH (septal and posterior wall thickness 1.8 cm) and arterial hypertension, particularly in older women, has been recognized four decades ago [94]. Diagnosis of LVOTO in patients with arterial hypertension is important because antihypertensive agents with vasodilating properties (vasodilating beta-blockers, angiotensin-converting enzyme, ACE, inhibitors, angiotensin receptor blockers, ARB, dihydropyridine calcium antagonists) can worsen LVOTO and associated symptoms and should be avoided if possible [3].

## Management

### Atrial fibrillation

The presence of AF is prevalent in approximately one out of five individuals with HCM and has been linked to considerable morbidity as well as mortality [95]. In particular, paroxysmal AF can cause acute worsening of HF in patients with both HF due to structural heart disease and intracavity obstruction. Contemporary management strategies have lowered the annual mortality rate attributable to AF to < 1% in HCM patients [96].

Outpatient Holter ECG is mandatory at initial assessment and during follow-up to screen for AF (see also Sect. 6.2).

Thromboembolic events are estimated to be prevalent in 27% of HCM patients with concomitant AF, with an incidence of approximately 4 per 100 patients [97]. Established stroke risk scores such as the CHA2DS2-VASc score underestimate the thromboembolic risk in this population and are therefore not applicable in HCM patients [96, 98]. Accordingly, thromboembolic prophylaxis with vitamin K antagonists or direct-acting oral anticoagulants is recommended in all HCM patients with clinical AF in whom oral anticoagulants are not contraindicated [3, 99]. Subclinical AF also increases stroke risk, with longer duration of episodes being associated with an increased thromboembolic risk [100, 101]. Anticoagulation is recommended in HCM patients with subclinical AF of > 24 h duration, and implementation of treatment should be evaluated in those with subclinical AF between 5 min and < 24 h duration [3]. Atrial flutter and AF do not differ with respect to thromboembolic risk [2].

Considering the significant thromboembolic risk in HCM patients with AF, screening to detect AF might be considered. In addition to established screening methods, such as opportunistic pulse palpation and outpatient ECG monitoring, commercially available wearable devices like smartwatches represent upcoming screening tools that will be used by an increasing number of consumers [99, 102, 103]. Caution is required when using these devices, as only few have been clinically evaluated, and even if AF is suspected, it needs to be confirmed by an ECG analyzed by a physician [99].

Given the compromised tolerance of AF in HCM patients, a rhythm control strategy is frequently the preferred choice for the management of this condition [2, 3]. Treatment with dofetilide or sotalol can suppress AF recurrence [3]. Although evidence from randomized controlled trials is lacking regarding the long-term prevention of AF relapses in HCM patients using antiarrhythmic drugs, observational studies suggest that amiodarone can successfully control AF recurrences [96, 104]. During long-term amiodarone treatment, regular screening for potential amiodarone side effects is mandatory. Thyrotoxicosis, in particular, can worsen HF symptoms. Percutaneous radiofrequency ablation represents a rhythm control strategy in HCM patients with symptomatic AF in whom antiarrhythmic drug treatment is ineffective or contraindicated, although data suggest that this procedure is less effective in HCM than in non-HCM patients regarding AF relapse [3, 105]. In patients with cavotricuspid isthmus-dependent atrial flutter, catheter ablation is an effective treatment option [99]. Concomitant surgical AF ablation might be considered in HCM patients with symptomatic AF undergoing open heart surgery, although data are sparse [3, 96]. Non-dihydropyridine calcium antagonists and/or beta-blockers are the favored rate control options in HCM patients [2, 3]. Due to the positive inotropic properties of digoxin, this drug remains an alternative rate control agent in non-obstructive HCM patients only.

### Management of left ventricular outflow tract obstruction

#### Definition

Resting obstruction is defined as a peak resting gradient of ≥ 30 mm Hg and is diagnosed in approximately one third of patients with HCM referred to tertiary care centers. Another third of HCM patients present with provocable (syn. “latent”) LVOTO, which is defined as a peak resting gradient < 30 mm Hg but ≥ 30 mm Hg during provocative maneuvers [3]. Significant LVOTO requiring treatment is defined as a peak LVOT gradient ≥ 50 mm Hg. The LVOTO is dynamic and dependent on ventricular load and myocardial contractility. Increases in myocardial contractility and/or decreases in LV preload or afterload increase the LVOT gradient [106, 107].

#### Clinical presentation

The LVOTO leads to diminished stroke volume and increased filling pressures. The extent of LVOTO increases during exercise and further during the immediate post-exercise recovery period [108]. Typical symptoms include exertional dyspnea, limited exercise capacity, angina pectoris and exertional syncope. Symptoms tend to worsen postprandially (within 1 h after meals), during conditions of dehydration (e.g., high temperatures, diarrhea) and with alcohol consumption.

#### Lifestyle modification

Obstructive HCM patients should avoid dehydration or exposure to extreme heat [2, 3]. Obesity is associated with increased LVOT gradients and an independent predictor of adverse outcomes in HCM patients [109–111]. Consequently, weight loss interventions may have the potential to lower the burden of morbidity and mortality in obese HCM patients with LVOTO. High-intensity and competitive sports are not recommended in patients with HCM and LVOTO [1].

#### Drug treatment

The LVOTO is associated with increased mortality, but no randomized controlled trial has yet been performed to assess the effect of LVOTO reduction on cardiovascular outcomes. Therefore, treatment of LVOTO is currently considered symptomatic. In asymptomatic patients, LVOTO may be treated if there is evidence of elevated filling pressures, or in a perioperative setting to minimize perioperative cardiovascular risk [112–114]. Cardiac myosin ATPase inhibitors or SRT are not indicated in asymptomatic patients [1, 2].

As LVOTO is a dynamic phenomenon and significantly modifiable based on alterations in preload and afterload, vasodilators such as phosphodiesterase 5 inhibitors and nitrates should be avoided in patients with LVOTO. Moreover, according to expert opinion, angiotensin-converting enzyme inhibitors, angiotensin receptor blockers and dihydropyridine calcium antagonists such as amlodipine should not be readily administered as these may increase the LVOT gradient [3].

Non-vasodilating selective beta-blockers (e.g., bisoprolol, metoprolol), titrated to a maximum tolerated dose, have been assigned a class I B recommendation by the current ESC guidelines to reduce the LVOT gradient and improve symptoms [1]. Alternatively, in the case of contraindications, intolerance or inefficacy of beta-blocker treatment, verapamil (alternatively diltiazem) is recommended [1]. In patients with a resting LVOT gradient ≥ 100 mm Hg, pulmonary hypertension or signs of cardiac congestion, verapamil or diltiazem should not be administered due to vasodilating properties and the risk of acute HF and pulmonary edema [3]. Combinations of beta-blocker and non-dihydropyridine calcium antagonist treatment increase the risk of bradycardia, conduction disorders and HF, and should only be prescribed in selected patients after careful evaluation [115]. If available, disopyramide in addition to a beta-blocker (or if this is not possible verapamil or diltiazem) is recommended in patients refractory to drug monotherapy. At present, disopyramide is not commercially available in Austria.

In severe LVOTO complicated by cardiogenic shock, norepinephrine and intravenous beta-blockers are recommended, while use of adrenaline can be deleterious due to its positive inotropic effects that further promote LVOT gradient increase.

Drug treatment of mid-ventricular and RV obstruction is equivalent to the treatment of LVOTO as described above.

##### Treatment of refractory symptoms

In patients with LVEF ≥ 55% who are symptomatic despite maximum tolerated drug treatment and in whom the maximum LVOT gradient remains ≥ 50 mm Hg, treatment with a cardiac myosin ATPase inhibitor (CMI) on top of pre-existing optimal treatment should be considered. In a randomized controlled trial, treatment with mavacamten after 30 weeks, as compared to placebo, significantly reduced the post-exercise LVOT gradient by 36 mm Hg and improved maximum oxygen consumption (peak VO _2_) by 1.4 ml/kg/min [116]. In another trial conducted in HCM patients referred for SRT who were treated with mavacamten over 16 weeks, only 18% remained eligible for SRT compared to 77% in the placebo group [117].

Initiation of CMI demands careful titration to the maximum tolerated dose guided by regular echocardiographic surveillance of LVEF and the LVOT gradient, because a dose-dependent and reversible decline in LVEF to less than 50% has been described in 5–10% of treated patients. The CYP2C19 genotyping is required before or during CMI initiation, because slow metabolizers should receive a maximum target dose of mavacamten of 5 mg rather than 15 mg. During the first 12 weeks of treatment, 4‑weekly TTE controls are called for, followed by 12-weekly TTE controls throughout treatment. The titration algorithm is complex and available in the product information. Given the high demand on both the care providers’ and patients’ resources, patient compliance must be considered before the initiation of treatment. At the time of this publication, mavacamten is the only CMI available and reimbursed in Austria, although aficamten could emerge as the second drug in this class.

Table 3 lists relevant available drugs for the treatment of LVOTO.

**Table 3:** Drugs approved in Austria for the treatment of cavity obstruction in hypertrophic cardiomyopathy

	Treatment initiation^a^	Maximum dose per day^a^	Half-life
*Beta-blockers*			
Bisoprolol	2.5 mg o.d.	5 mg b.i. d. 10 mg in total	10–12 h
Metoprolol	47.5 mg o.d.	95 mg b.i. d. 190 mg in total	1–9 h
Nebivolol	2.5 mg o.d.	5 mg b.i. d. 10 mg in total	10–50 h
*Non-dihydropyridine calcium antagonists*			
Verapamil	40 mg t.i. d.	160 mg t.i. d. 480 mg in total	3–7 h
Diltiazem	60 mg t.i. d.	120 mg t.i. d. 360 mg in total	4–10 h
*Cardiac myosin ATPase inhibitors*			
Mavacamten	2.5 mg o.d. or 5 mg o.d.	15 mg (5 mg^b^) o.d.	6–9 days (23 days^b^)

*b.i.* *d.* twice daily, *o.d.* once daily, *t.i.* *d.* three times daily

^a^ Under consideration of contraindications and potential side effects

^b^ In low CYP2C19 metabolizers

#### Septal reduction therapy

**Fig. 6:**
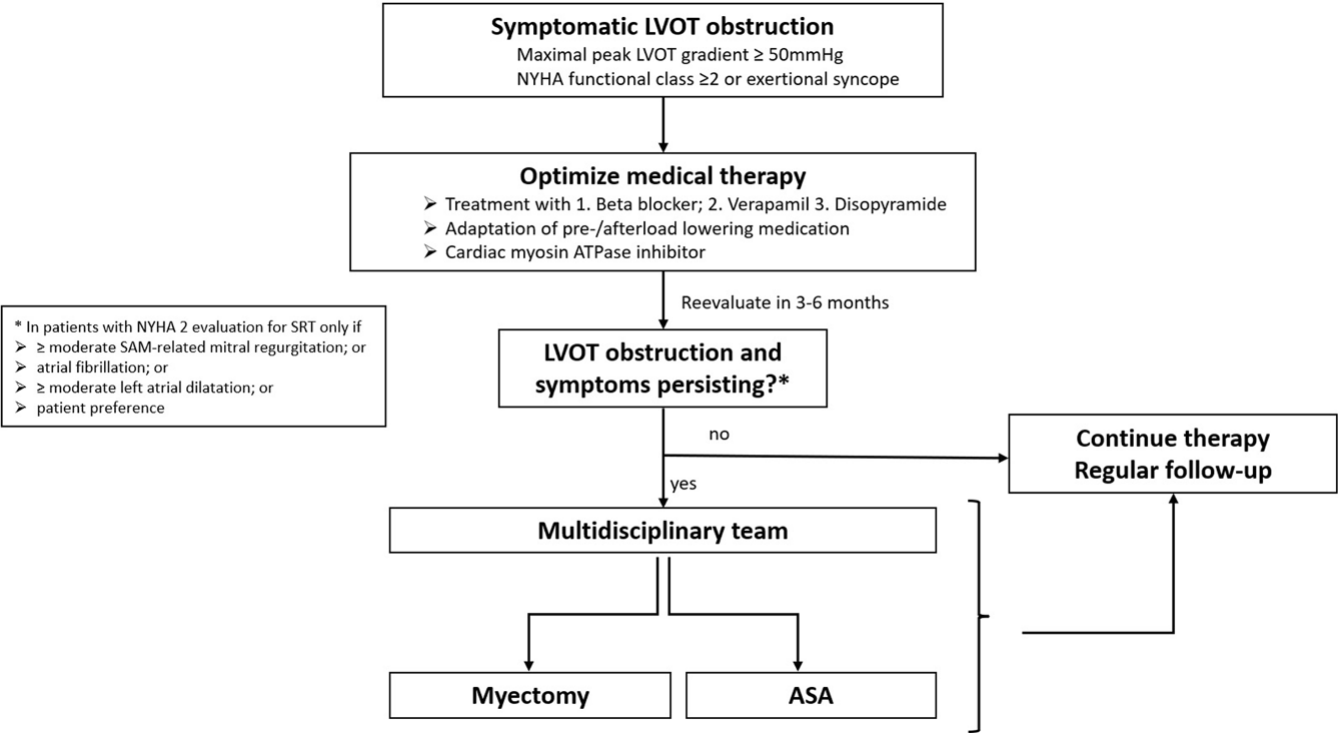
Management of left ventricular outflow tract obstruction. *ASA* alcohol septal ablation, *LVOT* left-ventricular outflow tract obstruction, *NYHA* New York Heart Association, *SAM* systolic anterior motion, *SRT* septal reduction therapy. (adapted from [1–3])

The SRT is recommended to improve symptoms in patients with a resting or maximum provoked LVOT gradient ≥ 50 mm Hg who are symptomatic with exertional dyspnea (at least NYHA functional class 2) or exertional syncope, despite maximum tolerated medical treatment including CMI. The SRT should be performed by experienced surgeons working in a multidisciplinary team with experience in the management of HCM. The treatment algorithm is depicted in Fig. 6.

##### Alcohol septal ablation

ASA was first described by Siegwarth et al. [118], who proposed septal myocardial mass reduction by targeted induction of myocardial necrosis. The procedure is performed via a transfemoral or transradial approach in the cardiac catheterization laboratory and requires both an interventionalist and an echocardiographer. ASA is performed with a back-up temporary pacemaker in place due to the 5–20% periprocedural risk of transient or permanent complete AV block. An over-the-wire balloon catheter is inflated in the septal branch in order to achieve complete occlusion. Echocardiographic contrast agent is administered through the lumen to confirm that contrast agent is staining at the very location of the intraventricular septum without contrasting papillary muscles, RV free wall or other locations of the left ventricle. Selection of the optimal septal branch is confirmed by radiocontrast injection proving the absence of any retrograde contrast leakage or antegrade collateralization. Approximately 0.1 ml of 95% ethanol per mm of septal thickness is slowly injected into the blocked septal branch. Considering the relevant thoracic pain caused by the iatrogenic myocardial necrosis, proper analgesia is mandatory. Intracoronary Injection of 95% ethanol leads to total occlusion of the septal branch vessel as well as complete destruction of the microvascular bed, leading to instant reduction of the LVOT gradient due to stunning of the myocardium; however, during the edematous phase of myocardial necrosis, the gradient can temporarily deteriorate compared to baseline, which is followed by stabilization at a lower value as scar formation is completed. After the procedure, the pacemaker has to be kept in place for at least 24 h. Serial measurements of creatinine kinase (CK) should be performed to obtain maximal CK levels reflecting the extent of induced myocardial necrosis. Telemonitoring is recommended during 48–72 h after the procedure to detect AV block. In more than 90% of patients treated with ASA, a peak LVOT gradient under provocation < 50 mm Hg can be achieved that is accompanied by clinical improvement of at least one NYHA functional class. Periprocedural mortality in large registries is less than 1%.

The most frequent complications are left or right bundle branch block and periprocedural AV block, necessitating the implantation of a permanent pacemaker in 10–20% [119]. In contrast to surgical myectomy, full success of ASA can be evaluated 3–12 months after the procedure due to remodelling after induced myocardial infarction, with subsequent formation of scar tissue. Staged ablation of another septal branch can be indicated in approximately 20% of patients in cases of persisting symptoms during follow-up due to relevant residual obstruction. In an observational registry including 952 patients, the 5‑year, 10-year, and 15-year survival rates were 95.8%, 88.3%, and 79.7%, respectively [120]. Even though there are no randomized clinical trials to confirm prognostic benefits, observational studies have demonstrated sustained symptom relief without increases in SCD risk or mortality [121–123]. An age-matched observational study by Liebregts et al. showed comparable prognosis across patients with non-obstructive HCM and patients with LVOTO who were treated with ASA [124]. Regarding the method of septal reduction, observational data suggest similar outcomes in terms of symptoms and mortality when comparing ASA with surgical myectomy [125]; however, it is noteworthy that no randomized controlled study has compared invasive treatment options to date.

##### Surgical myectomy

Surgical treatment of LVOTO has been conceptualized by Morrow and Braunwald at the end of the 1950s and has been considered the only effective therapeutic mode to relieve symptoms of LVOTO for decades.

From a pathophysiological standpoint, the trigger of progressive symptoms in patients with LVOTO is related to obstruction due to the anterior displacement of the distal part of the anterior mitral leaflet (SAM). The anterior mitral leaflet is continuously exposed to mechanical stress, which leads to thickening of the normal valve tissue. Accordingly, the pathophysiology of LVOTO is mostly characterized by bulging of the septum and thickening of the tissue of the anterior leaflet and the corresponding secondary chords. For this reason, the surgical correction of LVOTO is based on two principles: the reduction of septal bulging by myectomy and the treatment of the anterior mitral leaflet. Additional anatomical aspects can further aggravate the SAM phenomenon, such as the presence of hypertrophic secondary chords or aberrant muscle bands, which do not allow the posterior movement of the leaflet during systole. On the other hand, in some cases the anterior displacement of the mitral leaflet coaptation may be related to a hypermobile anterior or long posterior leaflet.

**Fig. 7:**
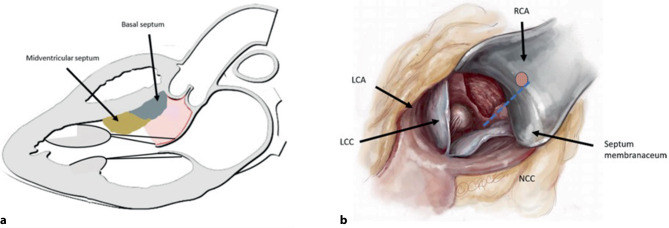
**a** Areas of interest for surgical treatment of left ventricular outflow tract obstruction. **b** Anatomical localization of septal myectomy through a transaortic access

The surgical treatment of LVOTO targets four different areas of interest (see Fig. 7):

1. The basal intraventricular septum (underneath the aortic valve),
2. The mid-ventricular septum (at the height between the free edge of the anterior mitral leaflet and the tip of the papillary muscles),
3. The anterior mitral leaflet itself and
4. The secondary chords as well as their origin from the papillary muscles.

A correctly performed septal myectomy includes the exact identification of septal bulging and the gradual en bloque resection of excessive tissue leftwards from the imaginary line between the right coronary ostium (to avoid AV node injury) and the anterolateral mitral commissure (muscular part of the distal intraventricular septum). If the surgical septum reduction is of adequate length and width, the reduction of LVOTO leads to a significant decrease of the subvalvular gradient in 80% of cases. In the remaining patients, mid-ventricular obstruction is present and treated by extended myectomy to this area. The treatment is completed by the complete mobilization of the anterior leaflet by resection of thickened secondary chords. In some cases, an aberrant muscle band connecting the anterior papillary muscle and the anterior mitral leaflet or tertiary chords to the septum can be found, which should be resected. Moving towards the LV apex, a misplaced hypertrophic papillary muscle towards the apical part of the intraventricular septum can be identified in some patients. The treatment is more complex here and may require a surgical approximation of the papillary muscles with several separate stitches. In cases with severe concentric LV hypertrophy in combination with either a long posterior or a hypermobile anterior mitral leaflet leading to SAM, mitral regurgitation can be effectively addressed by the Alfieri-stich. This is a surgical edge-to-edge repair by means of a simple suture-based connection between the A2 and P2 segments, which is performed through the transaortic access. This results in a relocation of the coaptation line towards the LV inflow tract. Mitral valve replacement is the ultima ratio in patients in whom all other treatment modalities regarding the mitral valve have failed.

Surgical myectomy is safe and effective, and the perioperative mortality in high-volume centers is low (0.5%) [92]. Clinical improvement (reduction of NYHA class ≥ 1) is reported in 90% of cases, with 75% of patients being completely asymptomatic after surgery if the procedure has been performed at an experienced center treating > 10 cases/year. Significant reduction of the LVOT gradient is the main criterion for a successful intervention and can be verified by intraoperative echocardiography. In principle, it is possible to extend the resection in a second pump run if the residual gradient is too high after the first correction. The reduction of LVOT gradients is maintained in the long run.

##### Multidisciplinary team

As detailed in the previous chapters, both the percutaneous and surgical approaches to achieve septum reduction have advantages and disadvantages. ASA might be also preferred in patients with high perioperative risk. Due to a lack of randomized, controlled head-to-head trials, there is no scientific evidence that generally supports one approach as the default approach. Meta-analyses of observational studies concluded that ASA and myectomy show globally comparable short-term and long-term effects regarding symptom improvement, mortality, occurrence of arrhythmia and SCD. ASA is associated with a higher re-intervention rate and higher risk of a periprocedural AV block necessitating permanent pacemaker implantation. LVOT gradient reduction appeared to be higher overall in patients undergoing myectomy [125–127].

Therefore, a patient-tailored strategy, defined by a multidisciplinary team and considering the patient’s clinical and anatomical characteristics as well as comorbidities, is key to a successful program. A comprehensive approach is illustrated in Fig. 6.

In cases of ideal septal coronary anatomy and more focal septal thickening, alcohol ablation can be an ideal first choice. On the other hand, in the presence of any other concomitant indication for open-chest surgery, such as valve disease or extensive coronary artery disease, concurrent myectomy should be favored. Surgical myectomy might also be the preferred choice in patients with high LVOT gradients ≥ 100 mm Hg indicating SRT, in cases of missing target septal branch(es), extreme thickening (> 30 mm) or extensive basal-to-apical septal thickening, or in cases of complex pathology of the mitral and/or subvalvular apparatus [128].

While strong evidence comparing the two procedures is lacking, it is the responsibility of the treating physicians to inform candidates objectively and in an unbiased manner about the pros and cons of the two procedures. Finally, patient preferences should be considered as well.

### Prevention of sudden cardiac death

HCM has repeatedly been identified as the most common cause of SCD in young people [36, 129]. According to emerging developments of ICDs over the last decades, the annual mortality rate in HCM is 0.5%, with a 5-year mortality rate of 2.5%. Major causes of death are SCD, HF and thromboembolism [17, 38]. The most common fatal arrhythmic events are ventricular tachycardia (VT) and ventricular fibrillation (VF), but even total atrioventricular blockage or pulseless electrical activity have been described [2].

#### Risk stratification, primary SCD prophylaxis

**Fig. 8:**
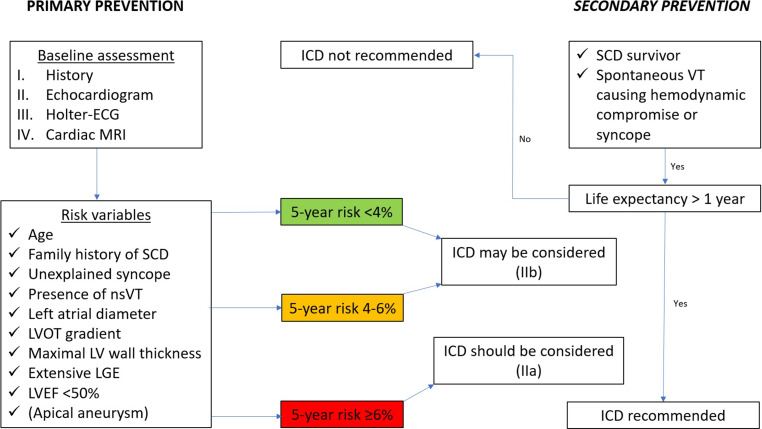
Indications for ICD implantation in patients with hypertrophic cardiomyopathy. *ECG* electrocardiogram, *ICD* implantable cardioverter defibrillator, *LGE* late gadolinium enhancement, *LV* left ventricular, *LVEF* left ventricular ejection fraction, *LVOT* left-ventricular outflow tract, *MRI* magnetic resonance imaging, *nsVT* non-sustained ventricular tachycardia, *SCD* sudden cardiac death, *VT* ventricular tachycardia. (adapted from [2, 3])

Evaluation of the SCD risk is a key issue in the management of patients with HCM. Clinical work-up on a regular basis at least once a year includes assessment of recent syncopes in addition to 24/48‑h outpatient ECG monitoring. Furthermore, TTE imaging is crucial in order to assess major features that are associated with an increased risk of SCD in adults according to recent guidelines [2]. If the calculated risk of SCD within the next 5 years is ≥ 6%, the primary prophylactic implantation of an ICD is recommended with a class IIa indication based on shared decision making (see Fig. 8). In cases of an intermediate (≥ 4–< 6%) or low risk (< 4%) of SCD, the presence of specific clinical features such as pronounced LGE on CMRI (> 15% of LV mass), LVEF < 50% or left apical aneurysms (as stated in the guidelines of the AHA) should be considered to guide ICD therapy on an individualized basis [1–3].

The 5‑year risk of SCD should be assessed at the initial evaluation and at follow-up visits conducted in 1–2-year intervals or at any change in clinical status [1, 3].

#### Secondary prophylaxis

An ICD implantation is recommended in survivors of cardiac arrest due to VF or VT and in patients with one episode of sustained VT who show a life expectancy > 1 year (see Fig. 8; [2, 3]).

### Heart failure

**Fig. 9:**
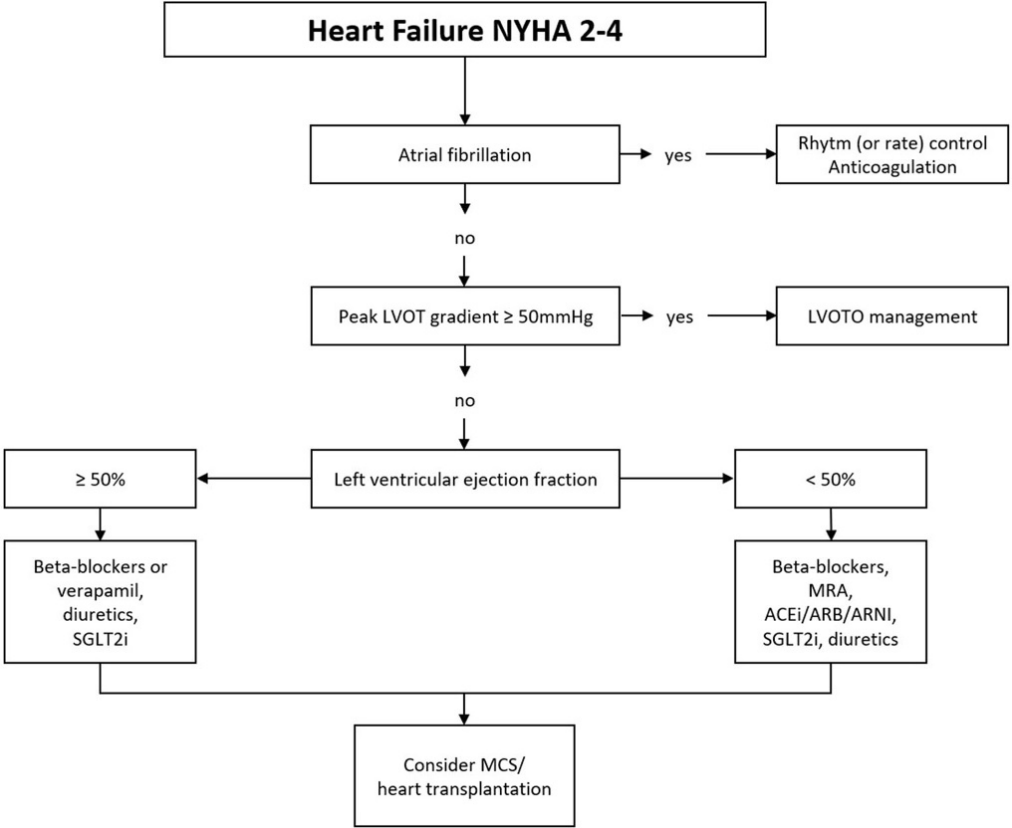
Management algorithm for patients with heart failure due to hypertrophic cardiomyopathy. *ACEi* angiotensin-converting enzyme inhibitor, *ARB* angiotensin receptor blocker, *ARNI* angiotensin receptor/neprilysin inhibitor, *LVOT* left ventricular outflow tract, *MCS* mechanical circulatory support, *MRA* mineralocorticoid receptor antagonist, *NYHA* New York Heart Association functional class, *SGLT2i* sodium-glucose co-transporter 2 inhibitor. (adapted from [1, 139, 140])

Symptoms of HF, such as exercise intolerance or dyspnea, are common in patients with HCM; however, the pathomechanism, prognosis and treatment options for patients with HF due to HCM differ significantly from those in patients with more common forms of HF (see Fig. 9; [36]). Diastolic dysfunction is an inherent feature of HCM that amplifies the critical importance of late diastolic filling by atrial contraction. Accordingly, the diagnosis and management of AF is a primary aspect of HF management in HCM. In patients with HCM and preserved LVEF ≥ 50%, HF is often caused by LVOTO, but it can also develop after successful treatment of LVOTO and in initially non-obstructive patients in the context of structural heart disease with worsening diastolic and systolic function. HCM patients with LVOTO develop NYHA stage III/IV in almost 40% (7% per year), while HCM patients without LVOTO develop NYHA stage III/IV in approximately 10% (1–2% per year; see also Sects. 4 and 5; [17]). Non-obstructive patients who are asymptomatic usually have good long-term prognosis, while those with signs and symptoms of HF can progress rapidly [130]. General determinants of progress towards HF include early diagnosis of HCM (age at diagnosis < 40 years), history of SRT (indicating previous LVOTO), female sex, presence of a pathogenic or likely pathogenic sarcomere mutation, left atrial dilation, AF, high serum levels of natriuretic peptides, extensive myocardial fibrosis, and progressive LV remodelling with a decline in LVEF below 50% [78, 130–133]. HCM phenocopies are associated with a significantly worse prognosis than HCM and must be recognized and treated early [134].

In HCM patients with HF due to LVOTO, treatment is focused on relieving LVOTO (see also Sect. 7.2). In non-obstructive HCM patients with HF and preserved ejection fraction, medical treatment is based on beta-blockers or calcium antagonists and diuretic therapy if needed. Clinical trials with pharmacological substances in HCM are often small, and HCM is underrepresented or not represented at all in HF trials [36, 135, 136]. Based on positive trial results, sodium-glucose co-transporter 2 inhibitors (SGLT2i) are indicated in HF across the whole LVEF spectrum and may also be used in symptomatic patients with non-obstructive HCM and LVEF ≥ 50% [137–139]. Whether CMI is useful in non-obstructive HCM with preserved ejection fraction is currently under investigation in clinical trials. Advanced HF due to HCM is defined as LVEF < 50% and should be treated according to the general principles of treatment of patients with HF and reduced ejection fraction that are based on beta-blockers, ACE inhibitors/ARB/angiotensin receptor/neprilysin inhibitors, mineralocorticoid receptor antagonists and SGLT2i [1, 17, 140].

In non-obstructive patients with HF and NYHA class ≥ 3, organ replacement treatment should be considered regardless of LVEF, but particularly if LVEF declines below 50% [1, 3, 136, 141–143]. Classical criteria for the diagnosis of advanced HF such as severely reduced LVEF, LV remodelling, low peak VO_2_ or recurrent HF hospitalizations are often not met; therefore, individual assessment in the multidisciplinary heart team is of great importance [136, 140].

Heart transplantation is a preferred treatment option for such patients. Long-term survival is probably superior to that of non-HCM patients, as affected patients are often relatively young and harbor only few comorbidities [144]. It is important to identify potential patients for heart transplantation at an early stage and to refer them to experienced centers.

Treatment with left ventricular assist devices (LVADs) has traditionally been viewed critically in patients with HCM/restrictive cardiomyopathy (RCM), as these patients often have restrictive LV filling and small LV dimensions. In fact, studies with LVAD have shown significantly worse results in the setting of HCM/RCM [145] and LVADs are only used in exceptional cases [142, 145, 146]. It remains to be seen whether total mechanical heart replacement with newer developments will play a role in the future, for example as a bridge to candidacy or to transplantation. In any case, early evaluation and, if necessary, listing for heart transplantation should not be delayed.

### Recommendations on physical exercise

Physical activity can be classified into recreational (i.e., regular low-to-moderate) activity and high-intensity/competitive activity. Recreational activity means engagement in sports for pleasure and leisure time activity, as opposed to an emphasis on performance and competition in high-intensity/competitive activity [1, 6].

Recommendations for physical activity in patients with HCM should be based on an individual risk assessment and shared decision making. Generally, low-to-moderate exercise is considered safe in patients with HCM and useful to improve physical capacity [147, 148], prevent cardiovascular disease, and maintain physical and psychological well-being [1, 6]. High-intensity and competitive sports, however, are associated with an increased risk of SCD and ventricular arrhythmias. Consequently, HCM patients with a history of SCD, unexplained syncope, exercise-induced arrhythmias, LVOTO or an ESC SCD risk score ≥ 4% should not perform high-intensity and competitive sports. Both HCM patients without any of these risk factors and HCM genotype-positive but phenotype-negative individuals may engage in high-intensity and competitive sports based on shared decision making [1, 6].

## Follow-up and lifetime management of HCM patients

**Fig. 10:**
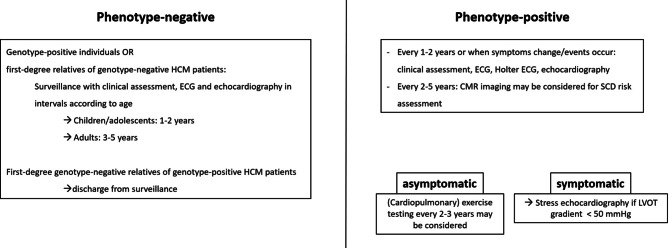
Follow-up of patients with hypertrophic cardiomyopathy. *CMR* cardiac magnetic resonance imaging, *ECG* electrocardiogram, *LVOT* left-ventricular outflow tract (adapted from [1–3])

Throughout their life, HCM patients are exposed to an increased risk of specific disease complications most of which require diagnosis and treatment at an HCM expert center. Therefore, lifelong follow-up is mandated to detect worsening of symptoms and ventricular function, LVOTO and AF, and to assess the risk of adverse events [1–3] (see Fig. 10).

### Echocardiography and cardiac magnetic resonance imaging

In patients with phenotype-positive HCM who are in a stable clinical condition and have had no event, TTE should be repeated every 1–2 years to assess the degree of myocardial hypertrophy, LVOTO, mitral regurgitation, and myocardial function. In patients who have experienced a change in clinical status or a recent clinical event, TTE should be repeated immediately [149].

Following ASA, TTE should be performed 3 and 12 months after the procedure to assess the result of the intervention [150]. In some patients, the effect of ASA on LVOTO can continue due to reverse remodelling. For screening purposes, TTE should be performed in first-degree relatives of patients with confirmed HCM as part of the initial family screening and periodic follow-up [151]. In individuals who are genotype-positive but phenotype-negative, repeated echocardiographic assessment is recommended at periodic intervals depending on age (1–2 years in children and adolescents, and 3–5 years in adults). Echocardiography should be performed when clinical status changes [152, 153]. In relatives of an index patient with positive genotype in whom the index patient’s mutation has been ruled out, no further screening and HCM-specific follow-up is recommended.

Repeated contrast-enhanced CMR imaging every 3–5 years for SCD risk stratification may be considered to assess changes in LGE and other morphological features associated with an increased risk of SCD including reduced LVEF, aneurysm formation at the LV apex (in case of mid-cavity obstruction), or progressive LV wall thickening [31].

### ECG and ECG monitoring

A 12-lead ECG is recommended as part of the periodic follow-up (every 1–2 years) in HCM patients with a stable clinical condition and without recent clinical events [154]. Outpatient ECG monitoring (24–48 h) should be performed as part of the periodic follow-up (every 1–2 years) to identify patients who are at high risk of SCD and to guide the management of arrhythmias [155].

In patients with new-onset palpitations or lightheadedness, extended outpatient ECG monitoring (48 h to 7 days) or event recording is recommended [156].

Patients with HCM at risk of AF (left atrial dilation, advanced age, NYHA III/IV HF) may be assessed by extended outpatient ECG as part of the periodic follow-up every 1–2 years. Additionally, extended outpatient ECG monitoring may be useful in HCM patients without risk factors for AF to screen for asymptomatic paroxysmal AF every 1–2 years [96].

### Cardiopulmonary exercise stress testing

Exercise stress testing may be considered every 2–3 years in HCM patients to assess functional capacity and/or symptom status.

Patients with non-obstructive HCM and advanced HF (NYHA functional class III–IV despite optimal guideline-directed management) should undergo cardiopulmonary exercise stress testing to assess and quantify the degree of functional impairment and for the selection of patients for mechanical circulatory support and/or heart transplantation [157].

### Genotype-positive, phenotype-negative individuals

In individuals who are genotype-positive and phenotype-negative for HCM, clinical assessment, ECG, and cardiac imaging are recommended at periodic intervals depending on age (every 1–2 years in children and adolescents, and every 3–5 years in adults) and whenever the patient’s clinical status changes [3, 153, 158].

## Gaps

1. Therapeutic options in non-obstructive HCM
  Heart failure in patients with non-obstructive HCM is often a result of increased LV filling pressures due to diastolic dysfunction, increased myocardial oxygen demand and microvascular dysfunction. This patient population has no proven medical treatment options, and heart transplantation is the only definitive treatment. CMI showed promising results in phase 2 studies and is currently under investigation.
2. Better understanding of mechanisms leading to genotype-negative HCM
  Only a subgroup of clinically diagnosed HCM patients has evidence of a (mono)genetic etiology. In these patients, determinants of HCM will likely prove to be multifactorial, not confined to the sarcomere, and involve environmental and/or other non-genetic factors. Intensive studies are required for better understanding of the various causes of HCM.
3. Improved diagnostic algorithms for rare HCM phenocopies
  The diagnosis of rare phenocopies of HCM, such as mitochondrial diseases or storage diseases, is challenging. Besides creating more awareness for these diseases among physicians, practicable algorithms with clear presentation of red flags and required diagnostic procedures are needed.
4. SCD prediction after septal reduction therapy
  Risk stratification in HCM is mandatory to identify patients at increased risk of SCD. Available risk calculators apply to patients with obstructive HCM. Whether the risk of SCD can be reduced by SRT is currently unclear. Early observations suggest a predictive role of atrial size [159]; however, larger studies are still pending.
5. Availability of randomized controlled trials
  Many recommendations in international guidelines are based on data from observational studies or expert opinion. More randomized controlled trials are warranted to prove and challenge recommended diagnostic and therapeutic concepts, particularly with respect to drug, device, interventional and surgical treatment.
6. Sodium-glucose co-transporter 2 inhibitors in HCM
  Patients with HCM were excluded from pivotal SGLTi HF trials. Whether these compounds are also effective in patients with HF due to HCM needs to be assessed in future studies.
7. Screening for atrial fibrillation
  AF is a common complication of HCM and can lead to thromboembolic events. Particularly paroxysmal AF often remains undiagnosed, because AF screening with traditional methods is limited by poor sensitivity. AF prediction scores are warranted to assess the indication of comprehensive AF screening in individual patients at high AF risk.
8. Antihypertensive therapy in obstructive HCM
  International guidelines recommend avoiding ACE inhibitors, ARB and amlodipine in patients with HCM and LVOTO. Therefore, LVOTO is often managed at the expense of adequate blood pressure control. There is an unmet need for randomized controlled trials to provide safe and effective blood pressure treatment regimens in patients with HCM and LVOTO.
9. Screening of coronary artery disease in patients with HCM
  HCM patients with concomitant advanced coronary artery disease (CAD) have a significantly worse prognosis when compared to HCM patients without severe CAD. As symptoms caused by HCM and CAD may be overlapping, clear recommendations for CAD screening (invasive angiography of coronary computed tomography) based on high-quality evidence are warranted.
